# Spatial analysis of tuberculosis treatment outcomes in Shanghai: implications for tuberculosis control

**DOI:** 10.4178/epih.e2022045

**Published:** 2022-05-01

**Authors:** Jing Zhang, Xin Shen, Chongguang Yang, Yue Chen, Juntao Guo, Decheng Wang, Jun Zhang, Henry Lynn, Yi Hu, Qichao Pan, Zhijie Zhang

**Affiliations:** 1 Department of Epidemiology and Biostatistics, School of Public Health, Fudan University, Shanghai, China; 2 Department of Population and Quantitative Health Science, School of Medicine, Case Western Reserve University, Cleveland, OH, USA; 3 Division of TB and HIV/AIDS Prevention, Shanghai Municipal Center for Disease Control and Prevention and Shanghai Institutes of Preventive Medicine, Shanghai, China; 4 Department of Epidemiology of Microbial Diseases, School of Public Health, Yale University, New Haven, CT, USA; 5 School of Epidemiology, Public Health and Preventive Medicine, Faculty of Medicine, University of Ottawa, Ottawa, ON, Canada; 6 Medical Science College, China Three Gorges University, Yichang, China

**Keywords:** Tuberculosis, Treatment outcome, Spatial analysis, Hospital effect, Physical accessibility, China

## Abstract

**OBJECTIVES:**

Tuberculosis (TB) treatment outcomes are a key indicator in the assessment of TB control programs. We aimed to identify spatial factors associated with TB treatment outcomes, and to provide additional insights into TB control from a geographical perspective.

**METHODS:**

We collected data from the electronic TB surveillance system in Shanghai, China and included pulmonary TB patients registered from January 1, 2009 to December 31, 2016. We examined the associations of physical accessibility to hospitals, an autoregression term, and random hospital effects with treatment outcomes in logistic regression models after adjusting for demographic, clinical, and treatment factors.

**RESULTS:**

Of the 53,475 pulmonary TB patients, 49,002 (91.6%) had successful treatment outcomes. The success rate increased from 89.3% in 2009 to 94.4% in 2016. The successful treatment outcome rate varied among hospitals from 78.6% to 97.8%, and there were 12 spatial clusters of poor treatment outcomes during the 8-year study period. The best-fit model incorporated spatial factors. Both the random hospital effects and autoregression terms had significant impacts on TB treatment outcomes, ranking 6th and 10th, respectively, in terms of statistical importance among 14 factors. The number of bus stations around the home was the least important variable in the model.

**CONCLUSIONS:**

Spatial autocorrelation and hospital effects were associated with TB treatment outcomes in Shanghai. In highly-integrated cities like Shanghai, physical accessibility was not related to treatment outcomes. Governments need to pay more attention to the mobility of patients and different success rates of treatment among hospitals.

## INTRODUCTION

Tuberculosis (TB) remains a major global health problem. It affects approximately 10 million people worldwide and kills more than 1 million every year [1,2]. In the early 1990s, the World Health Organization declared TB a global health emergency [3], and recommended the directly observed treatment short-course (DOTS) strategy [4]. The Stop TB Strategy and the End TB Strategy were launched in 2006 and 2015, respectively, to reduce the TB-related global burden, including the numbers of new cases and deaths [5-7]. In the 1990s, China started to implement the DOTS strategy, which covered 13 provinces and about half the country’s population [8], and was expanded to cover the entire country by 2005. The prevalence of smear-positive TB in China decreased from 170 cases per 100,000 in 1990 to 59 cases in 2010 [9]. However, China remains a high-TB-burden country, and in 2017, 9% of global TB incident cases occurred in this country [2].

The treatment success rate is one of the most important indicators for monitoring the implementation of the End TB Strategy [10]. The global rate was 82% in 2016, whereas the End TB Strategy targets a rate of ≥ 90% by 2025. Since TB treatment outcomes are a key programmatic output of any TB control program [11] and are very important for both public health and clinical perspectives [12], the determinants of treatment outcomes have been investigated worldwide [13,14]. Individual characteristics such as old age, cavitation on chest radiography, and TB treatment history have frequently been found to be related to poor treatment outcomes [15,16]. However, spatial information, such as patients’ residence location, public transportation, and the treatment hospital, which are related to the accessibility and quality of services [17], is often ignored.

Therefore, the objective of this study was to use an 8-year TB surveillance dataset in Shanghai, China to identify the relationships between TB treatment outcomes and spatial factors, focusing on physical accessibility, spatial autocorrelation, and hospital effects. We hope to provide additional insights into the TB surveillance system, particularly from a geographical perspective, based on the 8-year TB surveillance dataset in Shanghai.

## MATERIALS AND METHODS

### Study site

Shanghai, a coastal city located in east China, is the largest industrial and commercial city in China [18], and one of the most highly integrated cities in the world according to Globalization and World Cities [19]. Shanghai, which consists of 15 districts (Supplementary Material 1), had a residential population of 24.197 million in 2016, and 40.5% of them were migrants [20].

### Data source

In Shanghai, all patients with suspected pulmonary TB symptoms are referred to designated TB hospitals for diagnosis, and the results are then reported to the Shanghai Municipal Center for Disease Control and Prevention (SCDC) [21]. An electronic TB surveillance system managed by the SCDC was launched in 2004, and this system includes demographic, clinical, microbiological, and treatment information of each pulmonary TB patient. The current analysis was based on data from this electronic TB surveillance system, and included diagnosed pulmonary TB patients between January 1, 2009 and December 31, 2016.

The longitude and latitude of each patient’s residence at the time of diagnosis and of each designated hospital were geocoded using Baidu Maps (Baidu, Beijing, China), which is a Chinese-language-based web geocoding tool. The geographic coordinate system was WGS 1984 and the projection coordinate system was Gauss Kruger-Xian 1980 _3_degree_GK_cm_120E.

### Definitions and data preparation

According to the guideline for implementing TB control programs in China [22], patients who had successful treatment outcomes were defined as those who were cured or had completed treatment. Patients who had poor treatment outcomes were defined as those who failed TB treatment, died, were lost to follow-up, or discontinued treatment because of side effects [16,22,23].

Patients were grouped into local and migrant categories. Migrant patients were people whose household registration status was not in Shanghai through the Chinese *hukou* system. TB patients were managed by the SCDC of each district in the form of full-course supervision (i.e., directly observed treatment during the full course), intensive-phase supervision (i.e., directly observed treatment during the intensive course, and management, which included strengthening education, dispensing drugs and home visiting, during the continuation phase), full-course management (i.e., strengthening education, dispensing drugs, and home visiting regularly during the full course), or self-administration [15,22]. Bacteriologically positive cases were those that were smear-positive for acid-fast bacillus testing or had positive culture results. The standard treatment regimen was defined according to the guideline for implementing TB control programs in China, which included 2H_3_R_3_Z_3_E_3_/4H_3_R_3_, 2HRZE/4HR, 2H_3_R_3_Z_3_E_3_S_3_/6H_3_R_3_E_3_, 2HRZES/6HRE, and other regimens [22,23]. Personalized regimens were those other than the standard regimens and were usually used among drug-resistant patients. The treatment hospital was the hospital where a patient was treated most recently, and the proportion of patients transferring among hospitals was very low. The distance between the patient’s residence and the hospital was calculated in terms of Euclidean distance.

### Statistical analysis

Firstly, basic characteristics of TB patients were compared between the successful and poor outcome groups. Proportions were used to describe the distribution of categorical variables, and the chi-square test was used to compare the distributions between the two groups.

Secondly, the proportion of treatment outcomes was calculated for each study year, and the proportion of poor treatment outcomes was compared among designated hospitals.

Thirdly, we used Kulldorff spatial scan statistics to detect spatial clusters for the proportion of poor treatment outcomes in each year. This method identifies a significant excess of cases within a moving circle that increases in size until it reaches an upper size limit. We set this maximum spatial cluster size to 30% of all patients. The likelihood ratio test was based on Bernoulli models, and p-values were obtained by Monte Carlo simulation with 999 replications.

Lastly, the final models were built to identify the effects of physical accessibility, spatial autocorrelation, and hospitals on TB treatment outcomes. The dataset was randomly divided into a training data set and a test data set with a ratio of 3:1, and models were first developed using the training data and then tested using test data. Univariate logistic regression was used to identify risk factors with a p-value of ≤ 0.10 for poor TB treatment outcomes, and those risk factors were then included in a multivariable logistic regression model with a backward elimination procedure. In separate models, we also used an autoregression term and random intercepts to account for spatial autocorrelation and hospital effects, respectively [24-26]. A total of 4 models were established including (1) a multivariable logistic model containing non-spatial variables and variables related to physical accessibility (model 1); (2) an autologistic model (model 2) that combined the variables in model 1 with an autoregression term, considering the spatial correlations of patients’ residential locations and TB treatment outcomes; (3) a random intercept logistic model that combined the variables in model 1 with random hospital effects (model 3); and (4) a spatial model that included both an autoregression term and random hospital effects (model 4). Models were compared using both the Akaike information criterion (AIC) and the area under the receiver operating characteristic curve (AUC), and the change in the AIC was used to decide the relative importance of the variables in the best model.

Statistical significance was defined at the 5% alpha level. The Kulldorff spatial scan statistical analysis was performed using SaTScan version 9.5 (Martin Kulldorff and Information Management Services Inc., Boston, MA, USA). Other data analyses were conducted in R version 3.4.0 (R Core Team, Vienna, Austria).

### Ethics statement

The collection of data from TB cases was part of a continuing public health investigation of an emerging outbreak determined by the National Health and Family Planning Commission. Hence, this study was exempt from institutional review board assessment.

## RESULTS

### Basic characteristics of tuberculosis patients

In 2009-2016, there were a total of 54,301 pulmonary TB cases in Shanghai. After excluding 19 imprisoned patients and 807 cases with missing treatment outcomes, we included 53,475 (98.5%) patients in our analysis. Of these 53,475 patients, 36,656 (68.5%) were male (Table 1). The median age of the patients was 39.9 years (interquartile range, 26.3 to 58.4). Among the patients, 24,411 (45.6%) were migrants, 5,474 (10.2%) were retreated patients. Furthermore, 48,629 (90.9%) were under full-course supervision, and 27,303 (51.1%) were bacteriologically positive. The distance from patients’ home to the hospital where they were treated was less than 5 km for 15,413 (28.8%) patients and more than 15 km for 14,281 (26.7%).

### Spatial and temporal distribution of treatment outcomes in 2009-2016

**Figure 1.**
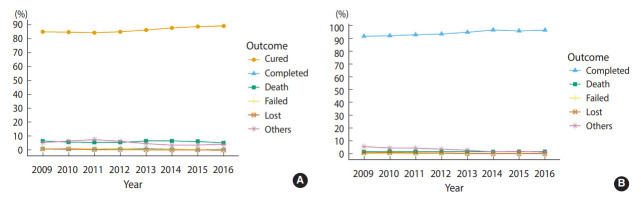
Treatment outcomes in pulmonary tuberculosis patients, 2009-2016. (A) In smear-positive pulmonary tuberculosis patients. (B) In smear-negative and unknown pulmonary tuberculosis patients. Others include those who discontinued treatment because of side effects.

Among the smear-positive patients (n=20,416), 17,596 (86.2%) patients were cured and 187 (0.9%) patients completed treatment, resulting in an overall successful treatment rate of 87.1% (95% confidence interval [CI], 85.8 to 88.4). In smear-negative or unknown patients (n=33,059), 31,219 (94.4%) completed treatment, corresponding to a successful treatment rate of 94.4% (95% CI, 93.4 to 95.5). Altogether, the successful treatment rate was 91.6% (95% CI, 90.8 to 92.5). During the 8-year period, the success rate increased significantly from 86.0% to 90.0% in smearpositive patients and from 91.8% to 96.6% in smear-negative or unknown patients (p<0.001) (Figure 1). The rates of death, treatment failure, and loss to follow-up remained relatively low.

**Figure 2.**
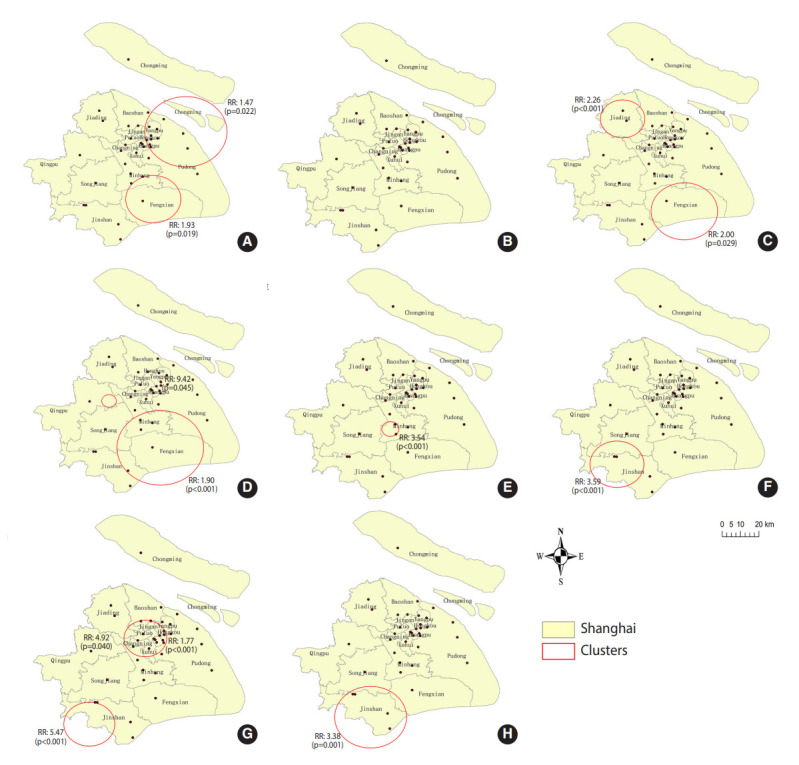
Annual spatial clusters of poor treatment outcomes in Shanghai from 2009 to 2016 (A) 2009, 2 spatial clusters, (B) 2010, 0 spatial clusters, (C) 2011, 2 spatial clusters, (D) 2012, 3 spatial clusters, (E) 2013, 1 spatial cluster, (F) 2014, 1 spatial cluster, (G) 2015, 2 spatial clusters, and (H) 2016, 1 spatial cluster. The red circles are the significant spatial clusters identified by the Kulldorff spatial scan statistic. The black dots are the 38 designated tuberculosis hospitals.

Spatial scan analysis indicated 12 significant spatial clusters for poor treatment outcomes (0-3 clusters for each study year) (Figure 2). The number of patients per cluster ranged from 7 to 1,835. The spatial clusters were mainly found in Minhang and Fengxian Districts in 2009 and 2011-2013, and in Jinshan District in 2014-2016. These districts are in suburban areas of Shanghai. During the study period, there were 38 designated hospitals in Shanghai, and the poor treatment outcome rate ranged from 2.2% to 21.4% for each hospital (Supplementary Material 2).

### Effects of spatial factors on treatment outcomes

The ethnic group, symptom to first diagnosis time, distance from home to hospital, and distance from home to the nearest subway line were not included in the multivariate models because of the variable selection procedure. Among the 4 multivariate models, when the autoregression term and random intercepts were considered, the AIC decreased from 19,638 in model 1 to 19,387 in model 4, and the AUC improved from 0.740 (model 1) to 0.761 (model 4) in the training dataset (Supplementary Material 3). Compared with model 3, model 4 had a statistically significantly lower AIC and a non-significantly changed AUC. Thus, model 4—the spatial model with both the autoregression term and random hospital effects—performed best among the 4 models.

Compared with model 1, model 4 resulted in similar risk factors that were associated with poor treatment outcomes: male sex (odds ratio [OR], 1.43, p<0.001); ≥ 45 years of age (OR, 1.34, p<0.001); an occupational status of laborer, retired, or house worker (OR, 1.23, p≤0.020), being a migrant (OR, 1.67, p<0.001), retreatment (OR, 2.17, p<0.001), management under intensive-phase supervision or self-administration (OR, 3.18, p<0.001), personalized regimen (OR, 1.54, p<0.001), bacteriological positivity (OR, 2.32, p<0.001), an interval of < 7 days from the first diagnosis to confirmed diagnosis (OR, 1.11, p=0.012), an interval of ≥7 days of confirmed diagnosis to treatment (OR, 1.67, p<0.001), and an earlier treatment year (OR, 0.88, p<0.001) (Table 2 and Supplementary Material 4).

From model 1 to model 4, the estimate for number of bus stops within 1 km from the patient’s home changed notably from a significant positive correlation to no correlation or even a negative correlation. In model 4, the autoregression term was highly significant (OR, 1.18, p<0.001). Management type, bacteriological status, age, treatment type, and treatment year were the top 5 important predictors (Supplementary Material 5), followed by the hospital effect, which showed an intra-class correlation of 0.077 (p<0.001). The autoregression term was ranked the 10th most important out of 14 variables. The number of bus stations around home was the least important variable in the model.

## DISCUSSION

Based on Shanghai TB surveillance data from 2009 to 2016, 91.6% of more than 50,000 pulmonary TB patients were successfully treated, which is comparable with China’s 93% success rate reported in 2016 [2]. The rate increased steadily over time, suggesting continuing improvement of TB control in Shanghai, and similar results were demonstrated in other studies [27,28].

In our spatial scan analysis, spatial clusters of poor treatment outcomes were detected, and most clusters were in districts with relatively weak economic status and sparse TB designated hospitals, such as Fengxian District (Figure 2 and Supplementary Material 1). Transportation in these areas was less convenient. The pattern of the clusters was accordant with previous studies showing that patients residing in resource-limited areas, such as rural areas, were more likely to have poor treatment outcomes [14,29]. We also found varying proportions of successful TB treatment outcomes among hospitals (ranging from 78.6 to 97.8%), and similar findings were reported by a study in southern Ethiopia, where the proportion of successful treatment varied from 70% to 94% across 8 health facilities [17]. There are various reasons for between-hospital differences, including different qualities of communication and relationships between patients and health workers [17,30,31], the heterogeneity of hospital service systems in delivering a package of interventions according to the needs of TB patients [32], and different medical practice styles [33]. In addition, the general financial status of the hospital and the pulmonary disease specialty, such as the proportion of pulmonary doctors and the percentage of budget allocated to pulmonary departments, could result in different proportions of successful treatment. Further research could focus on investigating the hospital factors related to the proportions of successful treatment.

Among the 4 logistic models, we found that model 4 (with the autoregression term and random hospital effects) fit the data the best. This suggests that additional spatial information can reduce information loss and improve the model’s predictive power. After adjusting for patient characteristics, the random hospital effects and autoregression term remained important predictors, indicating that the effect of spatial factors was non-negligible. In addition, the significance of the autoregression term suggested that there were other unmeasured variables that caused the spatial autocorrelation. For example, to compensate for the lack of information on the socio-economic status of the patients and the residential areas, we used patients’ occupations as a surrogate for income, but this might not have been adequate. The significance of random hospital effects suggests that different hospitals have different effects on treatment outcomes after adjusting for patients’ characteristics. Prospective studies might need to find the reason for this difference.

Our study showed that an increase in the number of bus stops within 1 km from home was no longer a protective factor after accounting for the random hospital effect, and even had a significantly negative effect on treatment outcomes. The reason for this is that if patients are treated at the same hospital, number of bus stops, which is an indicator of physical accessibility, is not related to the treatment outcomes, and the significant negative effect could be due to the extremely well-developed traffic and heavy population density in the neighborhood. When we constructed model 4 with just one hospital, such as Shanghai Pulmonary Hospital, which treats half of the patients in Shanghai and has a sufficient sample size, we found that the number of bus stops was not significantly associated with treatment outcomes (results not shown). This indicates that when patients are treated at the same hospital, physical accessibility to this hospital may not be associated with treatment outcomes in our study.

**Figure 3.**
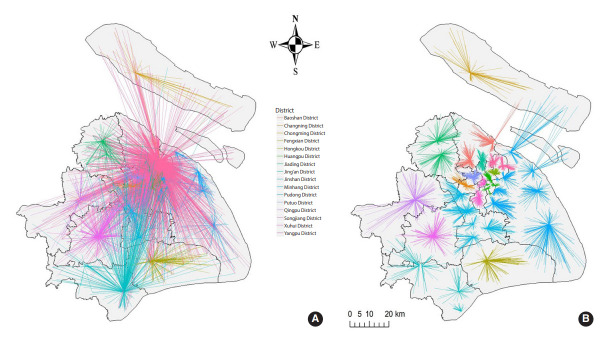
Straight-line distance from the residential addresses to the tuberculosis hospitals of pulmonary tuberculosis patients in Shanghai in 2009-2016. (A) Distance from the residential address to the treatment hospital. (B) Distance from the residential address to the nearest designated tuberculosis hospital. For legibility, only patients in 2016 were used to draw this figure.

The distance from home to the treatment center, which is another indicator of physical accessibility, was associated with treatment outcomes in previous studies [34,35] conducted in Ethiopia and India, but not in our study. This may be due to differences in economic development between Shanghai and other study sites. For example, Ethiopia is a predominantly rural society and 21 (28.6%) patients discontinued treatment because the TB clinic was too far from home. In contrast, only 13,707 (25.8%) patients in Shanghai chose to go to the nearest designated TB hospital and 14,062 (26.4%) patients chose to travel an additional 10 km to visit other designated hospitals (Figure 3). We would propose the idea of “latent space” to explain this behavior. Factors such as transportation, landscape, health status, and physical distance form the latent space. For people with poor economic status, places with convenient public transportation are closer to them. For people with higher income, places with better quality and services are closer to them. For people living near a subway station, places that they travel to using that subway line are closer. Thus, in the latent space, physical distance interacting with other factors forms a new concept of farness and proximity when people decide to travel from one place to another.

**Figure 4.**
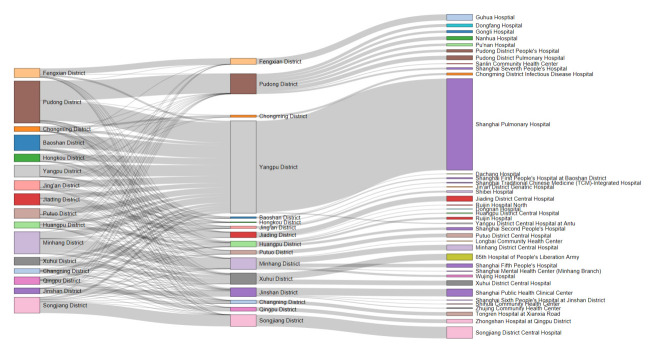
Flow of tuberculosis patients from the district of the residential address to the district of the treatment hospital, and to the treatment hospital in Shanghai, 2009-2016. A Sankey diagram was used, with the thickness of each link representing the number of patients.

To increase the physical accessibility of TB hospitals and improve the quality of patient management, the government of Shanghai has set at least 1 TB designated hospital in each district according to the national TB control plan since 2002 (Figure 4). Districts with large populations and areas, such as Pudong District and Minhang District, have more than 5 TB designated hospitals. However, 88.4%, 83.9%, 71.0%, and 60.5% of patients residing in Hongkou District, Baoshan District, Jing’an District, and Putuo District, respectively, went to Shanghai Pulmonary Hospital in Yangpu District. In total, Shanghai Pulmonary Hospital treated about half (47.7%) of all the patients in Shanghai. Due to differences in TB staff numbers, regular monitoring, and waiting time between these designated hospitals [36], patients typically choose a hospital for treatment based on their experience and preconceptions, rather than physical distance. The mobility of TB patients in the city can elevate the risk of TB transmission when patients use public transportation [37]. From 2009 to 2016, the number of patients treated at the Shanghai Pulmonary Hospital increased from 2,324 (35.6%) to 3,769 (59.4%), and this may be associated with increases in patients’ mobility and transmission risk. Measures, including health education and improvements in service quality and performance of all designated TB hospitals, should be taken to reduce the mobility of TB patients and the transmission risk.

There are several limitations of our study. First, other potential variables related to treatment outcomes (e.g., drug resistance status, body mass index, smoking, alcohol drinking, diabetes, and HIV status among others) [14,38-40] were not evaluated in the present study, and these variables could be the underlying reasons leading to the significance of the spatial autocorrelation and hospital effect. Therefore, more individual factors should be considered in future research. Second, the straight-line distance from home to hospital and the number of bus stops near the patient’s home may not be the best indicators of physical accessibility. For example, for patients in Chongming District (an island), the travel distance is much longer than the straight-line distance because of the limited number of bridges and ferries. Furthermore, the number of bus stops around home is a complex variable, which is also related to population density and economic development. Third, our findings are likely related to the degree of the area’s development, and may not be generalizable to other cities or areas with different economic characteristics.

In conclusion, we identified spatial clusters of poor treatment outcomes in suburban regions and spatial factors in association with TB treatment outcomes in Shanghai. Spatial autocorrelation and hospital effects need to be taken into consideration when risk factors for poor treatment outcomes are investigated. In highly-integrated cities like Shanghai, physical accessibility is not related to treatment outcomes. Governments need to pay more attention to the mobility of patients and different success rates of treatment among hospitals.

## Figures and Tables

**Table 1.** Basic characteristics of pulmonary tuberculosis patients with successful and poor treatment outcomes

Characteristics	Total	Successful	Poor	p-value^1^
Total	53,475 (100)	49,002 (91.6)	4,473 (8.4)	
Sex				<0.001
Male	36,656 (68.5)	33,195 (90.6)	3,461 (9.4)	
Female	16,819 (31.5)	15,807 (94.0)	1,012 (6.0)	
Ethnic group				0.023
Han	52,952 (99.0)	48,546 (91.7)	4,406 (8.3)	
Minorities	487 (0.9)	432 (88.7)	55 (11.3)	
Missing	36 (0.1)	24 (66.7)	12 (33.3)	
Age (yr)				<0.001
<30	18,703 (35.0)	17,661 (94.4)	1,042 (5.6)	
31-44	11,193 (20.9)	10,553 (94.3)	640 (5.7)	
45-59	11,428 (21.4)	10,524 (92.1)	904 (7.9)	
≥60	12,150 (22.7)	10,263 (84.5)	1,887 (15.5)	
Missing	1 (0.0)	1 (100)	0 (0.0)	
Occupation				<0.001
Clerks	6,171 (11.5)	5,897 (95.6)	274 (4.4)	
Laborers	13,929 (26.0)	12,703 (91.2)	1,226 (8.8)	
Retired and house workers	17,963 (33.6)	15,900 (88.5)	2,063 (11.5)	
Others and unknown	15,412 (28.8)	14,502 (94.1)	910 (5.9)	
Residence type				<0.001
Local	29,064 (54.4)	26,437 (91.0)	2,627 (9.0)	
Migrant	24,411 (45.6)	22,565 (92.4)	1,846 (7.6)	
Treatment type				<0.001
Initial treatment	48,001 (89.8)	44,602 (92.9)	3,399 (7.1)	
Retreatment	5,474 (10.2)	4,400 (80.4)	1,074 (19.6)	
Management type				<0.001
Full-course supervision	48,629 (90.9)	44,819 (92.2)	3,810 (7.8)	
Intensive-phase supervision	462 (0.9)	321 (69.5)	141 (30.5)	
Full-course management	4,110 (7.7)	3,758 (91.4)	352 (8.6)	
Self-administration	120 (0.2)	30 (25.0)	90 (75.0)	
Missing	154 (0.3)	74 (48.1)	80 (51.9)	
Bacteriological result				<0.001
Bacteriologically negative	18,045 (33.7)	17,270 (95.7)	775 (4.3)	
Bacteriologically positive	27,303 (51.1)	24,171 (88.5)	3,132 (11.5)	
Unknown	8,127 (15.2)	7,561 (93.0)	566 (7.0)	
Regimen type				<0.001
Standard regimens	30,034 (56.2)	28,072 (93.5)	1,962 (6.5)	
Personalized regimens	23,420 (43.8)	20,912 (89.3)	2,508 (10.7)	
Missing	21 (0.0)	18 (85.7)	3 (14.3)	
Symptom to first diagnosis time (day)				<0.001
<7	18,519 (34.6)	17,062 (92.1)	1,457 (7.9)	
7-29	23,042 (43.1)	21,154 (91.8)	1,888 (8.2)	
≥30	11,750 (22.0)	10,635 (90.5)	1,115 (9.5)	
Missing	164 (0.3)	151 (92.1)	13 (7.9)	
First diagnosis to confirmed diagnosis (day)				<0.001
<7	18,040 (33.7)	16,253 (90.1)	1,787 (9.9)	
7-29	27,807 (52.0)	25,728 (92.5)	2,079 (7.5)	
≥30	7,612 (14.2)	7,007 (92.1)	605 (7.9)	
Missing	16 (0.0)	14 (87.5)	2 (12.5)	
Confirmed diagnosis to treatment (day)				<0.001
<0	9,209 (17.2)	8,430 (91.5)	779 (8.5)	
0-6	42,952 (80.3)	39,493 (91.9)	3,459 (8.1)	
≥7	1,125 (2.1)	953 (84.7)	172 (15.3)	
Missing	189 (0.4)	126 (66.7)	63 (33.3)	
Distance from home to hospital (km)				-
<5	15,413 (28.8)	14,163 (91.9)	1,250 (8.1)	
5-9	14,970 (28.0)	13,669 (91.3)	1,301 (8.7)	
10-14	8,523 (15.9)	7,848 (92.1)	675 (7.9)	
≥15	14,281 (26.7)	13,069 (91.5)	1,212 (8.5)	
Missing	288 (0.5)	253 (87.8)	35 (12.2)	
Distance from home to the nearest subway line (km)				-
<2	15,159 (28.3)	13,816 (91.1)	1,343 (8.9)	
2-3	16,407 (30.7)	15,067 (91.8)	1,340 (8.2)	
4-5	8,795 (16.4)	8,088 (92.0)	707 (8.0)	
≥6	13,114 (24.5)	12,031 (91.7)	1,083 (8.3)	
No. of bus stops within 1 km from home				-
0-9	12,700 (23.7)	11,508 (90.6)	1,192 (9.4)	
10-39	14,200 (26.6)	13,134 (92.5)	1,066 (7.5)	
40-89	13,566 (25.4)	12,487 (92.0)	1,079 (8.0)	
≥90	13,009 (24.3)	11,873 (91.3)	1,136 (8.7)	

Values are presented as number (%); The percentages in the “total” column are column percentages; The percentages in the “successful” and “poor” columns are row percentages.

1 Mmissing results were not included in the tests.

**Table 2.** Independent risk factors for poor tuberculosis treatment outcomes in the univariate logistic model, model 1, and model 4

Characteristics	Univariate	p-value	Model 1	p-value	Model 4	p-value
Sex						
Male	1.00 (reference)		1.00 (reference)		1.00 (reference)	
Female	0.61 (0.56, 0.67)	<0.001	0.71 (0.65, 0.77)	<0.001	0.70 (0.64, 0.77)	<0.001
Ethnic group						
Han	1.00 (reference)		-	-	-	-
Minorities	1.31 (0.92, 1.82)	0.120	-	-	-	-
Age (yr)						
<30	1.00 (reference)		1.00 (reference)		1.00 (reference)	
31-44	0.94 (0.83, 1.06)	0.290	0.87 (0.76, 0.98)	0.028	0.86 (0.76, 0.98)	0.025
45-59	1.39 (1.25, 1.56)	<0.001	1.33 (1.17, 1.50)	<0.001	1.34 (1.18, 1.52)	<0.001
≥60	3.06 (2.79, 3.36)	<0.001	2.68 (2.35, 3.05)	<0.001	2.52 (2.21, 2.88)	<0.001
Occupation						
Clerks	1.00 (reference)		1.00 (reference)		1.00 (reference)	
Laborers	2.11 (1.80, 2.48)	<0.001	1.39 (1.17, 1.65)	<0.001	1.23 (1.03, 1.47)	0.020
Retired and house workers	2.83 (2.43, 3.31)	<0.001	1.45 (1.23, 1.72)	<0.001	1.48 (1.25, 1.76)	<0.001
Others and unknown	1.33 (1.13, 1.57)	0.001	1.16 (0.98, 1.38)	0.087	1.15 (0.97, 1.36)	0.116
Residence type						
Local	1.00 (reference)		1.00 (reference)		1.00 (reference)	
Migrant	0.84 (0.78, 0.90)	<0.001	1.63 (1.47, 1.79)	<0.001	1.67 (1.51, 1.85)	<0.001
Treatment type						
Initial treatment	1.00 (reference)		1.00 (reference)		1.00 (reference)	
Retreatment	3.12 (2.85, 3.41)	<0.001	2.12 (1.92, 2.34)	<0.001	2.17 (1.96, 2.40)	<0.001
Management type						
Full-course supervision	1.00 (reference)		1.00 (reference)		1.00 (reference)	
Intensive-phase supervision	4.70 (3.66, 6.00)	<0.001	4.28 (3.27, 5.55)	<0.001	3.18 (2.41, 4.19)	<0.001
Full-course management	1.07 (0.93, 1.23)	0.307	0.99 (0.86, 1.15)	0.945	0.94 (0.81, 1.09)	0.384
Self-administration	39.53 (24.70, 66.04)	<0.001	46.68 (28.51, 79.57)	<0.001	49.85 (29.76, 83.51)	<0.001
Regimen type						
Standard regimens	1.00 (reference)		1.00 (reference)		1.00 (reference)	
Personalized regimens	1.74 (1.61, 1.87)	<0.001	1.49 (1.37, 1.62)	<0.001	1.54 (1.41, 1.69)	<0.001
Bacteriological result						
Bacteriological negative	1.00 (reference)		1.00 (reference)		1.00 (reference)	
Bacteriological positive	2.78 (2.53, 3.06)	<0.001	2.33 (2.11, 2.57)	<0.001	2.32 (2.09, 2.56)	<0.001
Unknown	1.57 (1.38, 1.79)	<0.001	1.65 (1.43, 1.89)	<0.001	1.65 (1.43, 1.90)	<0.001
Symptom to first diagnosis time (day)						
<7	1.00 (reference)		-	-	-	-
7-29	1.01 (0.93, 1.10)	0.780	-	-	-	-
≥30	1.24 (1.12, 1.36)	<0.001	-	-	-	-
First diagnosis to confirmed diagnosis (day)						
<7	1.00 (reference)		1.00 (reference)		1.00 (reference)	
7-29	0.75 (0.70, 0.82)	<0.001	0.89 (0.82, 0.96)	0.004	0.90 (0.82, 0.98)	0.012
≥30	0.79 (0.70, 0.88)	<0.001	0.86 (0.76, 0.97)	0.014	0.90 (0.79, 1.02)	0.099
Confirmed diagnosis to treatment (days)						
<0	1.00 (reference)		1.00 (reference)		1.00 (reference)	
0-6	1.00 (0.91, 1.11)	0.967	0.95 (0.86,1.06)	0.381	1.04 (0.92, 1.17)	0.565
≥7	2.26 (1.83, 2.78)	<0.001	1.75 (1.40, 2.19)	<0.001	1.67 (1.32, 2.11)	<0.001
Year of registration	0.89 (0.87, 0.90)	<0.001	0.88 (0.87, 0.90)	<0.001	0.88 (0.86, 0.89)	<0.001
Distance from home to hospital (km)						
<5	1.00 (reference)		-	-	-	-
5-9	1.08 (0.98, 1.19)	0.105	-	-	-	-
10-14	0.99 (0.88, 1.11)	0.837	-	-	-	-
≥15	1.04 (0.95, 1.15)	0.392	-	-	-	-
Distance from home to the nearest subway line (km)						
<2	1.00 (reference)		-	-	-	-
2-3	0.88 (0.96, 0.80)	0.006	-	-	-	-
4-5	0.88 (0.98, 0.78)	0.025	-	-	-	-
≥6	0.90 (0.99, 0.81)	0.028	-	-	-	-
No. of bus stops within 1 km from home						
0-9	1.00 (reference)		1.00 (reference)		1.00 (reference)	
10-39	0.83 (0.92, 0.75)	<0.001	0.85 (0.77, 0.95)	0.004	0.97 (0.86, 1.09)	0.603
40-89	0.88 (0.98, 0.80)	0.017	0.86 (0.76, 0.96)	0.007	1.05 (0.93, 1.19)	0.459
≥90	0.97 (1.07, 0.88)	0.560	0.92 (0.82, 1.03)	0.169	1.14 (1.00, 1.30)	0.047
Autoregression term	1.34 (1.27, 1.41)	<0.001	-	-	1.18 (1.12, 1.26)	<0.001

Values are presented as odds ratio (95% confidence interval); All the results come from models developed by the training dataset.

## SUPPLEMENTARY MATERIALS

Supplementary Material 1. Population and GDP of each district in Shanghai in 2016

Supplementary Material 2. Successful and poor treatment outcome in 38 designated hospitals in Shanghai

Supplementary Material 3. Performance of different models in identifying risk factors for poor tuberculosis treatment outcome

Supplementary Material 4. AIC changes in the autologistic and random intercept spatial model

Supplementary Material 5. Results for the sensitivity analysis
