# Supplementary material for: Spatial analysis of tuberculosis treatment outcomes in Shanghai: implications for tuberculosis control
Source: Epidemiol Health. 2022 May 1;44:e2022045. doi: 10.4178/epih.e2022045 (PMC9684007; doi:10.4178/epih.e2022045)
Supplement: Supplementary Material 1. — Population and GDP of each district in Shanghai in 2016 [file epih-44-e2022045-suppl1.docx]

Supplementary Material 1. Population and GDP of each district in Shanghai in 2016

| District | GDP (in billion CNY) | Population (in ten thousand) | GDP per capita (CNY) |
| --- | --- | --- | --- |
| Huangpu | 201.92 | 65.86 | 306589 |
| Changning | 131.62 | 69.11 | 190450 |
| Pudong | 873.25 | 574.49 | 159500 |
| Jing’an | 166.22 | 107.41 | 154752 |
| Xuhui | 152.22 | 108.91 | 139766 |
| Yangpu | 162.95 | 131.52 | 123897 |
| Jiading | 187.59 | 156.81 | 119628 |
| Jinshan | 92.29 | 79.81 | 115637 |
| Hongkou | 88.98 | 80.94 | 109933 |
| Minhang | 210.12 | 253.79 | 82792 |
| Qingpu | 93.97 | 120.91 | 77718 |
| Putuo | 97.39 | 128.81 | 75607 |
| Fengxian | 72.72 | 115.99 | 62695 |
| Songjiang | 104.04 | 176.02 | 59106 |
| Baoshan | 104.87 | 202.29 | 51841 |
| Chongming | 31.17 | 69.61 | 44778 |

^1^GDP = Gross domestic product, CNY = Chinese Yuan
