# Supplementary material for: Spatial analysis of tuberculosis treatment outcomes in Shanghai: implications for tuberculosis control
Source: Epidemiol Health. 2022 May 1;44:e2022045. doi: 10.4178/epih.e2022045 (PMC9684007; doi:10.4178/epih.e2022045)
Supplement: Supplementary Material 2. — Successful and poor treatment outcome in 38 designated hospitals in Shanghai [file epih-44-e2022045-suppl2.docx]

Supplementary Material 2. Successful and poor treatment outcome in 38 designated hospitals in Shanghai

| Hospital name | Total  *n. (%)* | Successful  *n. (%)* | Poor  *n. (%)* |
| --- | --- | --- | --- |
| Shanghai Mental Health Center (Minhang Branch) | 112 (0.2) | 88 (78.6) | 24 (21.4) |
| Shanghai Public Health Clinical Center | 2043 (3.8) | 1714 (83.9) | 329 (16.1) |
| Jiading District Central Hospital | 1441 (2.7) | 1234 (85.6) | 207 (14.4) |
| Zhujing Community Health Center | 221 (0.4) | 190 (86.0) | 31 (14.0) |
| Wujing Hospital | 577 (1.1) | 496 (86.0) | 81 (14.0) |
| Shanghai Sixth People's Hospital at Jinshan District | 72 (0.1) | 62 (86.1) | 10 (13.9) |
| Shihua Community Health Center | 97 (0.2) | 84 (86.6) | 13 (13.4) |
| Longbai Community Health Center | 107 (0.2) | 94 (87.9) | 13 (12.1) |
| Pudong District Pulmonary Hospital | 1125 (2.1) | 989 (87.9) | 136 (12.1) |
| 85th Hospital of People's Liberation Army | 1780 (3.3) | 1567 (88.0) | 213 (12.0) |
| Guhua Hosptial | 1694 (3.2) | 1491 (88.0) | 203 (12.0) |
| Jin'an District Geriatric Hospital | 110 (0.2) | 98 (89.1) | 12 (10.9) |
| Chongming District Infectious Disease Hospital | 568 (1.1) | 507 (89.3) | 61 (10.7) |
| Shanghai Traditional Chinese Medicine (TCM)-Integrated Hospital | 187 (0.4) | 168 (89.8) | 19 (10.2) |
| Zhongshan Hospital at Qingpu District | 1062 (2.0) | 961 (90.5) | 101 (9.5) |
| Shanghai Fifth People's Hospital | 977 (1.8) | 884 (90.5) | 93 (9.5) |
| Minhang District Central Hospital | 1502 (2.8) | 1362 (90.7) | 140 (9.3) |
| Ruijin Hospital | 563 (1.1) | 513 (91.1) | 50 (8.9) |
| Huangpu District Central Hospital | 248 (0.5) | 227 (91.5) | 21 (8.5) |
| Shanghai Seventh People's Hospital | 499 (0.9) | 457 (91.6) | 42 (8.4) |
| Pudong District People's Hospital | 707 (1.3) | 651 (92.1) | 56 (7.9) |
| Shanghai Pulmonary Hospital | 25523 (48.0) | 23525 (92.2) | 1998 (7.8) |
| Shanghai First People's Hospital at Baoshan District | 310 (0.6) | 286 (92.3) | 24 (7.7) |
| Nanhua Hospital | 976 (1.8) | 901 (92.3) | 75 (7.7) |
| Ruijin Hospital North | 100 (0.2) | 93 (93.0) | 7 (7.0) |
| Shanghai Second People's Hospital | 681 (1.3) | 635 (93.2) | 46 (6.8) |
| Yangpu District Central Hospital at Antu | 288 (0.5) | 271 (94.1) | 17 (5.9) |
| Putuo District Central Hospital | 1034 (1.9) | 975 (94.3) | 59 (5.7) |
| Dongfang Hospital | 805 (1.5) | 761 (94.5) | 44 (5.5) |
| Dachang Hospital | 93 (0.2) | 88 (94.6) | 5 (5.4) |
| Xuhui District Central Hospital | 1330 (2.5) | 1263 (95.0) | 67 (5.0) |
| Tongren Hospital at Xianxia Road | 982 (1.8) | 934 (95.1) | 48 (4.9) |
| Dongnan Hospital | 86 (0.2) | 82 (95.3) | 4 (4.7) |
| Songjiang District Central Hospital | 3262 (6.1) | 3132 (96.0) | 130 (4.0) |
| Shibei Hospital | 514 (1.0) | 496 (96.5) | 18 (3.5) |
| Pu'nan Hospital | 684 (1.3) | 663 (96.9) | 21 (3.1) |
| Gongli Hospital | 647 (1.2) | 631 (97.5) | 16 (2.5) |
| Sanlin Community Health Center | 182 (0.3) | 178 (97.8) | 4 (2.2) |

Percentages in the “Total” column are column percentages. Percentages in the “Successful” and “Poor” columns are row percentages.
