# Supplementary material for: Spatial analysis of tuberculosis treatment outcomes in Shanghai: implications for tuberculosis control
Source: Epidemiol Health. 2022 May 1;44:e2022045. doi: 10.4178/epih.e2022045 (PMC9684007; doi:10.4178/epih.e2022045)
Supplement: Supplementary Material 3. — Performance of different models in identifying risk factors for poor tuberculosis treatment outcome [file epih-44-e2022045-suppl3.docx]

Supplementary Material 3. Performance of different models in identifying risk factors for poor tuberculosis treatment outcome

| Model number | Model description | Number of variables | AIC | AUC (Train data) | AUC (Test data) |
| --- | --- | --- | --- | --- | --- |
| Model 1 | Multivariate model | 12 | 19638 | 0.7402 (0.7313-0.7492) | 0.7383 (0.7222-0.7545) |
| Model 2 | Autologistic model | 13 | 19550 | 0.7456 (0.7369-0.7544) * | 0.7275 (0.7114-0.7436) * |
| Model 3 | Random intercept model | 13 | 19414 | 0.7596 (0.7511-0.7681) * | 0.7452 (0.7292-0.7611) * |
| Model 4 | Autologistic and random intercept model | 14 | 19387 | 0.7606 (0.7521-0.7690) † | 0.7429 (0.7270-0.7588) † |

* *P* value<0.05 comparing with Model 1

† *P* value>0.05 comparing with Model 3
