# Supplementary material for: Spatial analysis of tuberculosis treatment outcomes in Shanghai: implications for tuberculosis control
Source: Epidemiol Health. 2022 May 1;44:e2022045. doi: 10.4178/epih.e2022045 (PMC9684007; doi:10.4178/epih.e2022045)
Supplement: Supplementary Material 4. — AIC changes in the autologistic and random intercept spatial model [file epih-44-e2022045-suppl4.docx]

Supplementary Material 4. AIC changes in the autologistic and random intercept spatial model

| Variable | AIC* | AIC change† | Rank‡ |
| --- | --- | --- | --- |
| Management type | 19704.75 | 317.87 | 1 |
| Bacteriological result | 19676.35 | 289.47 | 2 |
| Age group | 19645.8 | 258.92 | 3 |
| Treatment type | 19597.58 | 210.70 | 4 |
| Year of Registration | 19585.47 | 198.59 | 5 |
| Random intercept term of hospitals | 19550.32 | 163.44 | 6 |
| Residence type | 19483.81 | 96.93 | 7 |
| Regimen type | 19473.3 | 86.42 | 8 |
| Sex | 19445.28 | 58.40 | 9 |
| Autoregression term | 19414.37 | 27.49 | 10 |
| Occupation | 19410.42 | 23.54 | 11 |
| Confirmed diagnosis to treatment | 19401.82 | 14.94 | 12 |
| First diagnosis to confirmed diagnosis | 19389.65 | 2.77 | 13 |
| Number of bus stations within 1 km from home | 19388.49 | 1.61 | 14 |

* AIC for the spatial model after removing the variable

† AIC change comparing to the autologistic and random intercept spatial model

‡ Rank for AIC change from large to small, the smaller the rank is, the more important the variable is.
