# Supplementary material for: Spatial analysis of tuberculosis treatment outcomes in Shanghai: implications for tuberculosis control
Source: Epidemiol Health. 2022 May 1;44:e2022045. doi: 10.4178/epih.e2022045 (PMC9684007; doi:10.4178/epih.e2022045)
Supplement: Supplementary Material 5. — Results for the sensitivity analysis [file epih-44-e2022045-suppl5.docx]

Supplementary Material 5. Results for the sensitivity analysis

| Characteristic | All  OR (95% CI) | All  *P* | Shanghai Pulmonary Hospital  OR (95% CI) | Shanghai Pulmonary Hospital  *P* | Other Hospitals  OR (95% CI) | Other Hospitals  *P* |
| --- | --- | --- | --- | --- | --- | --- |
| Sex |  |  |  |  |  |  |
| Male | Ref. |  | Ref. |  | Ref. |  |
| Female | 0.70 (0.64-0.77) | <0.001* | 0.76 (0.67-0.87) | <0.001* | 0.66 (0.58-0.75) | <0.001* |
| Age group |  |  |  |  |  |  |
| <30 | Ref. |  | Ref. |  | Ref. |  |
| 31-44 | 0.86 (0.76-0.98) | 0.025* | 0.96 (0.77-1.19) | 0.725 | 0.83 (0.70-0.97) | 0.018* |
| 45-59 | 1.34 (1.18-1.52) | <0.001* | 1.74 (1.44-2.11) | <0.001* | 1.10 (0.93-1.31) | 0.255 |
| ≥60 | 2.52 (2.21-2.88) | <0.001* | 3.13 (2.54-3.86) | <0.001* | 2.31 (1.94-2.75) | <0.001* |
| Occupation |  |  |  |  |  |  |
| Clerks | Ref. |  | Ref. |  | Ref. |  |
| Laborers | 1.23 (1.03-1.47) | 0.020* | 1.28 (0.97-1.69) | 0.087 | 1.21 (0.96-1.53) | 0.109 |
| Retired and Houseworkers | 1.48 (1.25-1.76) | <0.001* | 1.45 (1.12-1.89) | 0.005* | 1.47 (1.16-1.86) | 0.001* |
| Others and unknown | 1.15 (0.97-1.36) | 0.116 | 1.14 (0.90-1.46) | 0.292 | 1.24 (0.96-1.60) | 0.098 |
| Residence type |  |  |  |  |  |  |
| Local | Ref. |  | Ref. |  | Ref. |  |
| Migrant | 1.67 (1.51-1.85) | <0.001* | 1.47 (1.26-1.71) | <0.001* | 1.84 (1.60-2.12) | <0.001* |
| Treatment type |  |  |  |  |  |  |
| Initial treatment | Ref. |  | Ref. |  | Ref. |  |
| Retreatment | 2.17 (1.96-2.40) | <0.001* | 2.53 (2.20-2.90) | <0.001* | 1.86 (1.60-2.15) | <0.001* |
| Management type |  |  |  |  |  |  |
| Full course supervision | Ref. |  | Ref. |  | Ref. |  |
| Intensive phase supervision | 3.18 (2.41-4.19) | <0.001* | 5.77 (3.36-9.70) | <0.001* | 2.61 (1.88-3.61) | <0.001* |
| Full course management | 0.94 (0.81-1.09) | 0.384 | 0.69 (0.56-0.84) | <0.001* | 1.30 (1.03-1.63) | 0.027* |
| Self-administration | 49.85 (29.76-83.51) | <0.001* | 26.02 (14.06-49.46) | <0.001* | 200.84 (59.93-673.15) | <0.001* |
| Regimen type |  |  |  |  |  |  |
| Standard regimens | Ref. |  | Ref. |  | Ref. |  |
| Personalized regimens | 1.54 (1.41-1.69) | <0.001* | 1.43 (1.25-1.64) | <0.001* | 1.64 (1.45-1.86) | <0.001* |
| Bacteriological result |  |  |  |  |  |  |
| Bacteriological negative | Ref. |  | Ref. |  | Ref. |  |
| Bacteriological positive | 2.32 (2.09-2.56) | <0.001* | 2.49 (2.11-2.94) | <0.001* | 2.27 (2.00-2.59) | <0.001* |
| Unknown | 1.65 (1.43-1.90) | <0.001* | 1.65 (1.36-2.00) | <0.001* | 2.12 (1.66-2.72) | <0.001* |
| First diagnosis to confirmed diagnosis (days) |  |  |  |  |  |  |
| <7 | Ref. |  | Ref. |  | Ref. |  |
| 7-29 | 0.90 (0.82-0.98) | 0.012* | 0.92 (0.81-1.04) | 0.173 | 0.88 (0.78-1.00) | 0.044* |
| ≥30 | 0.90 (0.79-1.02) | 0.099 | 0.76 (0.63-0.93) | 0.007* | 1.00 (0.84-1.18) | 0.982 |
| Confirmed diagnosis to treatment (days) |  |  |  |  |  |  |
| <0 | Ref. |  | Ref. |  | Ref. |  |
| 0-6 | 1.04 (0.92-1.17) | 0.565 | 0.87 (0.71-1.05) | 0.145 | 1.11 (0.95-1.29) | 0.178 |
| ≥7 | 1.67 (1.32-2.11) | <0.001* | 1.36 (0.88-2.06) | 0.155 | 1.84 (1.38-2.44) | <0.001* |
| Year of Registration | 0.88 (0.86-0.89) | <0.001* | 0.85 (0.83-0.87) | <0.001* | 0.90 (0.87-0.92) | <0.001* |
| Distance from home to hospital (km) |  |  |  |  |  |  |
| <5 | Ref. |  | Ref. |  | Ref. |  |
| 5-9 | 1.06 (0.96-1.18) | 0.245 | 0.91 (0.77-1.08) | 0.262 | 1.14 (0.99-1.3) | 0.071 |
| 10-14 | 1.06 (0.93-1.2) | 0.383 | 0.9 (0.74-1.1) | 0.306 | 1.16 (0.97-1.38) | 0.101 |
| ≥15 | 1.26 (1.11-1.43) | <0.001 | 1.04 (0.87-1.26) | 0.656 | 1.45 (1.23-1.72) | <0.001 |
| Number of bus stops within 1 km from home |  |  |  |  |  |  |
| 0-9 | Ref. |  | Ref. |  | Ref. |  |
| 10-39 | 0.97 (0.86-1.09) | 0.603 | 0.95 (0.78-1.16) | 0.637 | 0.97 (0.84-1.12) | 0.651 |
| 40-89 | 1.05 (0.93-1.19) | 0.459 | 1.03 (0.86-1.25) | 0.739 | 1.02 (0.85-1.21) | 0.844 |
| ≥90 | 1.14 (1.00-1.30) | 0.047* | 1.05 (0.87-1.26) | 0.613 | 1.18 (0.98-1.43) | 0.088 |
| Autoregression term | 1.18 (1.12-1.26) | <0.001* | 1.37 (1.25-1.49) | <0.001* | 1.05 (0.97-1.15) | 0.241 |

^1^* *P* values <0.05

^2^All the results come from models developed by the training dataset.

^3^OR= odds ratio, CI= confidence interval
